# Comparative analysis of therapeutic efficiency and costs (experience in Bulgaria) of oral antidiabetic therapies based on glitazones and gliptins

**DOI:** 10.1186/s13098-015-0059-7

**Published:** 2015-07-16

**Authors:** Elena Pavlova Filipova, Katya Hristova Uzunova, Toni Yonkov Vekov

**Affiliations:** Science Department, Tchaikapharma High Quality Medicines, Inc, 1 G.M. Dimitrov Blvd, 1172 Sofia, Bulgaria; Department of Medical ethics, management of health care and information technology, Medical University, Pleven, Bulgaria

**Keywords:** Type 2 diabetes, Pioglitazone, Gliptins, Efficiency, Safety

## Abstract

Type 2 diabetes mellitus is a serious, chronic, progressive and widespread disease. Metformin is the most commonly prescribed initial therapy, but combination with other antidiabetic agents usually becomes necessary due to the progression of the disease. Pioglitazone is recommended as a second-line therapy because of its strong antihyperglycemic effect and its ability to reduce insulin resistance. Treatment with pioglitazone is associated with a significantly lower risk of cardiovascular complications and hypoglycemia, while simultaneously improving the lipid profile and the symptomatic and histological changes in the liver. Gliptins (sitagliptin and vildagliptin) are a new class of oral antidiabetic drugs which reduce glycated hemoglobin by a different mechanism. Although the efficacy of sitagliptin and vildagliptin is close to that of pioglitazone, the lack of long-term safety data and the higher price question their predominant use. The objective of this review is to highlight the advantages of mono- and combination therapy with pioglitazone in comparison with gliptins and to underline the inconsistencies in the medicinal and reimbursement policy in Bulgaria.

## Introduction

 Type 2 diabetes mellitus (T2DM) is a global epidemic with an estimated worldwide prevalence of 246 million people (6 %) in 2007 and the number is expected to reach 380 million (7.3 %) by 2025 [1]. As a chronic disease with serious consequences, T2DM requires costly therapy and care. Metformin is the most commonly prescribed first-line therapy in the world, but due to progressive deterioration of blood glucose control during the natural progression of the disease, combination therapy usually becomes a necessity.

Thiazolidinediones (TZDs), such as pioglitazone, are a class of antidiabetic drugs that exert their action by binding to peroxisome proliferator-activated receptor gamma (PPAR-γ) [2]. Peroxisome proliferator-activated receptors (PPARs) are expressed in various tissues: kidney, heart, muscle, adipose tissue (PPAR-α); heart, muscle, colon, kidney, pancreas, spleen, macrophages, white adipose tissue (PPAR-γ); brain, adipose tissue and skin (PPAR-δ) [3]. The primary role of PPAR-γ appears to be in regulating glucose and lipid metabolism along with adipogenesis. PPAR-γ is thought to enhance the actions of insulin [4]. TZDs bind specifically with PPAR-γ and by activating these receptors they perform a variety of biological functions such as the reduction of insulin resistance, which is responsible for their application as anti-hyperglycaemic agents. Gliptins are a new oral drug class for the treatment of T2DM which reduce blood sugar (glucose) levels by a different mechanism. They inhibit the enzyme dipeptidyl peptidase-4 (DPP-4), thereby increasing the circulating levels of incretins (gut hormones that enhance insulin secretion). Sitagliptin and vildagliptin belong to this drug class.

 According to the therapeutic guidelines of the National Institute for Health and Care Excellence (NICE) glitazones are recommended as second-line therapy in patients who fail to achieve adequate glycemic control (glycated hemoglobin (HbA_1c_) ≥ 6.5 %) after administration of metformin and/or sulphonylureas, especially if the person has marked insulin sensitivity [5]. Gliptins are also recommended as a second line of therapy, and may be preferable to TZDs in case of problems with weight gain or if the TZD (pioglitazone) is contraindicated [5].

The American Diabetes Association (ADA) and the European Association for the Study of Diabetes (EASD), in their updated guideline (2015), suggest TZDs and gliptins as second-line therapy strategies if metformin is not sufficient for achieving a satisfying HbA_1c_ level [6].

 Other strategies offered by both guidelines include glucagon-like peptide-1 receptor agonists, sodium glucose co transporter-2 inhibitors, sulphonylureas, insulin [5, 6].

Gliptins (sitagliptin, vildagliptin) in combination with metformin are predominantly used in therapeutic practice in Bulgaria. Sales of sitagliptin/metformin for 2014 in the country amounted to BGN 13 million (EUR 6,646,794), and of vildaglliptin/metfomin – to BGN 12.5 million (EUR 6,391,149) according to IMS (Intercontinental Marketing Services) Health. Sales of pioglitazone were BGN 0.1 million (EUR 51,129).

The established disproportion in sales is due to the different levels of reimbursement of medicinal products - sitagliptin - 100 %, vildagliptin - 100 %, pioglitazone - 25 %, sitagliptin/metformin - 50 %, vildaglliptin/metfomin - 50 %. Decisions for the inclusion of drugs in the Positive Drug List (PDL) and determination of the levels of reimbursement are made by the National Council on Prices and Reimbursement of Medicinal Products (NCPRMP).

This is a comparative analysis of the efficacy, safety and costs of oral antidiabetic therapies based on gliptins and glitazones, aiming to assess the objectivity of the decisions concerning pricing and reimbursement based on pharmacoeconomic evidence presented by the pharmaceutical industry.

A search of PubMed, Medline, Sciencedirect, Food and Drug Administration (FDA), European Medicines Agency (ЕМА), NCPRMP websites was performed with the following keywords: TZDs, pioglitazone, DPP-4 inhibitors, gliptins, sitagliptin, vildagliptin, metformin, efficacy, safety, adverse reactions, and combination therapy. Original articles and reviews as well as abstracts discussing the efficacy and safety of pioglitazone, sitagliptin and vildagliptin mono- or combination therapy, published in the 2000–2015 period were included in the current review.

## Review

### Efficacy of pioglitazone

TZDs favorably influence upon the majority of the components of insulin resistance characteristic of T2DM, like adiposity, dyslipidaemia, hyperglycaemia, hypertension, cardiovascular abnormalities, hyper coagulation, vasculopathy, accelerated atherosclerosis, and changes in liver and ovaries [7]. TZDs, pioglitazone in particular, successfully reduce HbA_1c_ (Table 1). Studies have shown decrease in HbA_1c_ of 0.92 % and 1.05 % compared to placebo for pioglitazone 15 mg and 30 mg, respectively [8, 9] and some authors even report values of 2 % [4]. When this parameter is considered, the hypoglycemic profile of pioglitazone resembles that of sulphonylurea and metformin unlike the gliptins for which some authors indicate a reduction of HbA_1c_ of 0.7 % to 0.9 % for sitagliptin [10, 11] and around 0.7 % for vildagliptin [12] in comparison with placebo. Other authors claim that sitagliptin and vildagliptin provide similar improvements in HbA_1c_ levels when compared with metformin, sulfonylureas or glitazones without contributing to weight gain and hypoglycemia [13] with reductions of HbA_1c_ of up to−1.0 % for sitagliptin and 0.9 % for vildagliptin [14].

**Table 1:** Comparative table of clinical trials investigating the efficacy of therapies based on glitazones and gliptins versus placebo

Reference	N	Treatment	Baseline values for HbA_1c_ [%]	Results		Statistical significance
				Glycated hemoglobin HbA_1c_ [%]	Fasting plasma glucose (FPG) [mmol/L]	
Lü et al., 2011 [8]	236	SU + PLB	In the range of 7.0 to 12.0	−0.28 ± 0.11	+0.17 ± 1.92	SS
		SU + PIO 30 mg		−0.92 ± 0.10	−1.48 ± 2.08	*p* < 0.05
Scherbaum et al., 2002 [9]	84	PLB + diet				SS
	89	PIO 15 mg + diet		−0.92	−1.9	
	78	PIO 30 mg + diet		−1.05	−2.0	
Yang et al., 2012 [10]	395	MET + PLB	8.5			SS
		MET + SIT		−0.9	−1.2	*p* < 0.001
Barzilai et al., 2011 [11]	206	PLB	7.8			SS
		SIT 50 (100) mg		−0.7	−1.5	*p* < 0.001
Yang et al., 2015 [12]	136	SU + PLB	8.6	−0.2		SS
	143	SU + VIL 100 mg	8.7	−0.7		*p* < 0.001
Lukashevich et al., 2014 [15]	160	PLB + MET + glimepiride	8.80	−0.25	+0.02	SS
	158	VIL + MET + glimepiride	8.75	−1.01	−1.11	*p* < 0.001

*EQW* exenatide, *MET* metformin, *PIO* pioglitazone, *SIT* sitagliptin, *VIL* vildagliptin, *SU* sulphonylurea, *PLB* placebo, *SS* statistically singnificant, *SN* statistically non-significant

It appears that TZDs reduce fasting plasma glucose (FPG) as well. Lü et al. have shown that pioglitazone is more effective in decreasing FPG when compared to placebo - - 1.48 mmol/L versus control group [8]. The reduction in comparison with placebo was 1.2 mmol/L [10] for sitagliptin and 1.11 mmol/L [15] for vildagliptin, respectively.

Results from trials conducted on both humans and animals have shown that TZDs preserve β-cell function, increase high-density lipoprotein (HDL) cholesterol and low-density lipoprotein (LDL) cholesterol with 10 to 15 % [16] and decrease triglyceride levels with the latter effect being more pronounced in the case of pioglitazone [4]. Some studies suggested that gliptins improve lipid parameters as well, with vildagliptin having a more beneficial effect on the lipid profile than sitagliptin [17].

### Efficacy of monotherapy with pioglitazone compared to monotherapy with sitagliptin and vildagliptin

The clinical trial DURATION-4 [18], which enrolled 820 subjects (696 completed all 26 weeks of therapy) aimed to monitor and assess the effectiveness of exenatide, metformin, pioglitazone and sitagliptin in patients with T2DM. The authors reported decrease in HbA_1c_ of 1.63 % in the pioglitazone group compared to 1.15 % in the sitagliptin group. The average value of HbA_1c_ at the end of the study was the lowest in the pioglitazone group−6.84 % for pioglitazone and 7.32 % to sitagliptin. The data was statistically significant and showed the notably greater effect of TZDs on the glycemic profile of patients. When FPG reduction was taken into account pioglitazone proved to be more efficient:−2.6 mmol/L for pioglitazone;−1.1 mmol/L for sitagliptin. Both drugs had a similar effect on the function of β-cells, but pioglitazone significantly improved insulin sensitivity compared to sitagliptin as measured by geometric mean HOMA-S (ratio of end point [last observation carried forward] to baseline): [+1.5 (0,06)] for pioglitazone and [+1.0 (0,04)] for sitagliptin.

Rosenstock et al. compared monotherapy with pioglitazone and monotherapy with vildagliptin and reported significantly greater effectiveness for pioglitazone. It reduced HbA_1c_ (1.4 ± 0.1 % reduction for monotherapy with pioglitazone versus 1.1 ± 0.1 % for vildagliptin) and FPG (1.9 ± 0.2 mmol/L reduction for pioglitazone versus 1.3 ± 0.2 mmol/L for vildagliptin) to a greater extent than the gliptins [19].

Pérez-Monteverde et al. reported results from a randomized double-blind trial comparing the efficacy of sitagliptin and pioglitazone monotherapy. Reductions in HbA_1c_ were similar in both groups -−1.0 % and−0.9 % for sitagliptin and pioglitazone, respectively. Both therapies reduced FPG similarly:−1.48 mmol/L and−1.56 mmol/L for sitagliptin and pioglitazone, respectively. It should be noted that while sitagliptin was given in its maximum recommended dose of 100 mg from the very beginning, pioglitazone was not - patients received 15 mg which were only later titrated to 30 mg [20] (Table 2).

**Table 2:** Comparative table of clinical trials investigating the efficacy of therapies based on glitazones versus gliptins

Reference	N	Treatment	Baseline values for HbA_1c_ [%]	Results		Statistical significance
				Glycated hemoglobin HbA_1c_ [%]	Fasting plasma glucose (FPG) [mmol/L]	
Pérez-Monteverde et al., 2011 [20]	492	SIT 100 mg		−1.0	−1.48	
		PIO 15 mg (30 mg)		−0.9	−1.56	
Russell-Jones et al., 2012 [18]	248	EQW 2 mg	Ranging from 8.0 to 8.6 across treatment groups	−1.53	−2.3	SS
	246	MET 2000 mg		−1.48	−2.0	
	163	PIO 45 mg		−1.63	−2.6	*p* < 0.001
	163	SIT 100 mg		−1.15	−1.1	
Rosenstock et al., 2007 [19]	161	PIO 30 mg	8.7 ± 1.0	−1.4 ± 0.1	−1.9 ± 0.2	SS
	154	VIL 100 mg	8.6 ± 1.0	−1.1 ± 0.1	−1.3 ± 0.2	
	144	PIO + VIL 15/50 mg	8.8 ± 0.9	−1.7 ± 0.1	−2.4 ± 0.2	
						*p* < 0.001
	148	PIO + VIL 30/100 mg	8.8 ± 0.9	−1.9 ± 0.1	−2.8 ± 0.2	
Chawla et al., 2013 [25]	52	SIT + MET 100/>1500 mg	8.076 ± 0.722	−0.656 ± 0.21	−1.1	NS *P* = 0.268 for the between group difference
		PIO + MET 30/>1500 mg	8.228 ± 0.822	−0.748 ± 0.35	−1.7	
Liu et al., 2013 [26]	60	SIT + MET + SU 100/≥1500/ half maximal dose		−0.71 ± 0.12	−1.27 ± 0.22	NS *p* = 0.16 between group difference for HbA_1c_
	59	PIO + MET + SU 30/≥1500/ half maximal dose		−0.94 ± 0.12	−1.98 ± 0.22	
Bergenstal et al., 2010 [27]	170	EQW 2 mg + MET	8.5	−1.5		SS *p* < 0.0001 for EQW vs. SIT; *p* = 0.0165 for EQW vs. PIO
	172	PIO 45 mg + MET		−1.2		
	172	SIT 100 mg + MET		−0.9		
Lee et al., 2013 [28]	31	MET + gliclazide or glimepiride	8.9	−2.5	From 9.24 to 5.71	SS *p* < 0.001 for difference in HbA_1c_from baseline for all groups
	30	MET + PIO 15 mg	8.8	−2.8	From 9.66 to 6.16	
	38	MET + SIT 100 mg	9.4	−2.7	From 9.60 to 5.86	
Blonde et al., 2009 [29]	1653	MET + VIL ≥1000/100 mg		−0.68 ± 0.02		SS
	825	MET + TZDs ≥1000 mg		−0.57 ± 0.03		*p* = 0.001
Bolli et al., 2008, 2009 [30, 31]	295	MET + VIL > 1500/100 mg	8.4 ± 1.0	−0.88 ± 0.5	−1.4 ± 0.1	SS
						*p* < 0.001 for all treatment groups at week 52 vs. baseline
	281	MET + PIO > 1500/30 mg	8.4 ± 0.9	−0.98 ± 0.06	−2.1 ± 0.1	
Kaur M. et al., 2014 [32]	30	MET + VIL 500/50 mg	8.43 ± 0.75	−0.75	−1.75	SS
						*p* < 0.001 for all treatment groups vs. baseline
	30	MET + PIO 500/15 mg	8.55 ± 0.84	−0.85	−1.85	
	30	VIL + PIO 50/15 mg	8.56 ± 0.69	−1.35	−2.6	
Jindal et al., 2015 [1]	30	MET + PIO 1000/30 mg		No values, the authors comment that there are not statistically significant differences in the reduction of HbA_1c_ between the two groups	No values, the authors comment that there are not statistically significant differences in the reduction of FPG between the two groups	SS
						*p* < 0.001 for all treatment groups vs. baseline
	30	MET + VIL 1000/100 mg				
Kaur K. et al., 2014 [33]	25	PIO 30 mg + MET + SU	10.93 ± 2.9	−1.65	−2.83	SS
						*p* < 0.001 for all treatment groups vs. baseline
	25	VIL 50 mg + MET + SU	11.3 ± 0.6	−1.23	−3.35	

*EQW* exenatide, *MET* metformin, *PIO* pioglitazone, *SIT* sitagliptin, *VIL* vildagliptin, *SU* sulphonylurea, *TZDs* Thiazolidinediones, *PLB* placebo, *SS* statistically singnificant, *SN* statistically non-significant

Reported data has indicated that the probability of reaching target HbA_1c_ < 7 % is higher in the case of therapy with pioglitazone [21].

### The efficacy of combination therapy pioglitazone + metformin as compared to the combination therapies sitagliptin + metformin and vildagliptin + metformin

Few therapeutic agents for the treatment of T2DM address both insulin resistance and β-cell function when administered as monotherapy. Pioglitazone and metformin increase insulin sensitivity and improve β-cell function, with TZDs having a more prominent effect on β-cells. These characteristics of the two drugs make their combination suitable either as initial therapy, or as very rapid add-on therapy for patients who do not achieve target glycemic control [22]. At the same time, gliptins (sitagliptin, vildagliptin) do not influence insulin resistance [23]. In preclinical studies, DPP-4 inhibitors have been shown to promote β-cell proliferation but there is no clinical evidence yet of the protective effects of incretin enhancers on β-cells in humans [24].

When comparing the combined therapy with pioglitazone and metformin with a combination of sitagliptin and metformin, a number of studies have demonstrated the advantage of combining the hypoglycaemic effects of metformin with those of TZDs (Table 2). Chawla et al. reported changes in levels of HbA_1c_ from baseline for sitagliptin (group 1)−0.656 ± 0.21 % and−0.748 ± 0.35 % for pioglitazone (group 2). After 16 weeks, the mean reductions in FPG were 1.09 mmol/L and 1.69 mmol/L, respectively, for group 1 and 2 [25]. In a randomized open-label study, Liu et al. observed mean change in HbA_1c_ from baseline−0.94 ± 0.12 % with pioglitazone and−0.71 ± 0.12 % with sitagliptin, which again confirmed the positive effect of TZDs. The mean change in FPG were−1.98 ± 0.22 mmol/L with pioglitazone and−1.27 ± 0.22 mmol/L with sitagliptin, with a difference between the two groups of−0.72 ± 0.32 mmol/L (*P* = 0.02) [26]. Bergenstal et al. compared the glycemic effect of pioglitazone and sitagliptin as adjunctive therapy to metformin in patients with T2DM in the study DURATION-2. Both therapies led to reduced levels of HbA_1c_, but it was more pronounced in the combination of pioglitazone and metformin: sitagliptin (−0.9 %, 95 % CI−1.1 to−0.7) and pioglitazone (−1.2 %, 95 % CI−1.4 to−1.0) [27]. Lee et al. have concluded that dual therapies using combinations of metformin and pioglitazone or sitagliptin show similar glycemic efficacy among patients with T2DM [28]. The percentage of reduction in HbA_1c_, observed by the authors has confirmed this statement (−2.8 % for pioglitazone with metformin;−2.7 % for sitagliptin with metformin), but it should be noted that while sitagliptin was taken at the maximum recommended dose of 100 mg, pioglitazone was not - patients received 15 mg while the maximum recommended dose is 45 mg.

The randomized, open-label, comparative study GALIANT, which assessed the effectiveness of treatment with vildagliptin compared to TZDs as adjunctive therapy to metformin (≥1000 mg/day) in patients with T2DM, showed that changes in HbA_1c_ levels in the two groups did not differ significantly. The authors concluded that the efficacy of both combination therapies is similar [29]. At the same time Bolli et al. found that pioglitazone in addition to metformin, is superior to vildagliptin + metformin, as it leads to significant reductions in HbA_1c_ (−0.98 ± 0.06 %) and FPG (−2.1 ± 0.1 mmol/L). In comparison, the values obtained for these parameters in the group treated with vildagliptin were−0.88 ± 0.5 % and−1.4 ± 0.1 mmol/L, respectively [30, 31]. The objective of a prospective, open, randomized, parallel study involving 90 patients was to determine the effectiveness and safety of three combinations of antihyperglycemic agents – metformin + pioglitazone; vildagliptin + metformin; vildagliptin + pioglitazone. After 12 weeks of therapy, HbA_1c_ fell by 0.85 % for metformin + pioglitazone and by 0.75 % for vildagliptin + metformin. A greater reduction of FPG was also reported in the group treated with pioglitazone. Significant reduction of insulin resistance was observed in all three groups, but when comparing metformin + pioglitazone and vildagliptin + metformin, the advantage was again in favor of the TZDs - 28.75 % against 18.76 % for the gliptins [32]. A recent (2015) multicenter, open label, randomized, parallel study comparing the effectiveness of vildagliptin and pioglitazone in combination with metformin, has shown that both drugs have similar efficacy in improving glycemic control in patients with T2DM for 24 weeks. There is a significant decrease in FPG in both groups. There are no statistically significant differences in the reduced levels of HbA_1c_ after 12 and 24 weeks between the two groups. Improvements in glycemic control influence favorably β-cell function in both groups [1]. When comparing triple therapies with metformin, sulphonylurea and pioglitazone or vildagliptin, Kaur et al. reported a significant decrease in HbA_1c_ in the TZDs group:−1.65 % in the comparison with−1.23 % for vildagliptin [33].

Results have indicated that the combination therapy with pioglitazone + metformin is more effective than sitagliptin + metformin, while the comparison with vildagliptin + metformin has shown that the two treatment regimens have similar efficacy.

### Safety of pioglitazone

T2DM is serious, chronic, progressive and widespread disease. In patients with T2DM, the risk of macrovascular complications (coronary artery disease, peripheral arterial disease, stroke) and microvascular complications (diabetic nephropathy, neuropathy and retinopathy) is high. In most of these patients metabolic disorders are observed, which themselves are significant risk factors. It is believed that the increased risk of cardiovascular complications is due to not only but also to lipid disorders, hypertension, chronic vascular inflammation and overall state of susceptibility to atherothrombosis in patients with diabetes. Patients with T2DM have an increased risk of fragility fractures despite increased body weight and normal or higher bone mineral density.

It is known that the efficiency of pioglitazone is associated with the implementation of good glycemic control and improvement of insulin sensitivity on the one hand; on the other hand it improves dyslipidemia, hypertension and microalbuminuria in patients with T2DM. Some studies have shown that pioglitazone increases the levels of HDL cholesterol and reduces the levels of triglyceride, fasting plasma free fatty acids, without affecting the levels of total cholesterol and LDL cholesterol [34, 35]. As specific agonists of the PPAR-γ, TZDs reduce the levels of circulating pro-inflammatory biomarkers of atherosclerosis [36], moreover, in patients treated with pioglitazone significantly lower rate of progression of coronary atherosclerosis was observed [37]. The effect of TZDs was associated with increased levels of adiponectin (vascular protective adipokine) and reduced levels of tissue necrosis factor α, which in turn leads to a decreased risk of cardiovascular complications. TZDs exert beneficial effect on coronary and peripheral vasodilation, with minimal improvement of blood pressure. Small controlled studies, using surrogate markers such as the intima-media thickness of the carotid artery, have shown improvements in patients treated with TZDs [38]. Protective effect against restenosis after percutaneous intervention in TZDs treated patients was also indicated [39, 40]. During the Duration-4 trial significant changes in serum lipids were not observed. The decrease in systolic blood pressure were:−1.3 mmHg (0.8 mmHg),−1.7 mmHg (1.0 mmHg), and−1.8 mmHg (1.0 mmHg) in therapy with exenatide, pioglitazone and sitagliptin, respectively. Reduction in diastolic blood pressure is achieved only on therapy with pioglitazone:−2.5 mmHg (0.6 mmHg). Compared with other drugs pioglitazone decreased heart rate [18].

ACT NOW study (for the prevention of diabetes) shows that pioglitazone leads to a reduction of the risk of developing of T2DM by 70 % in addition to ensuring stable glycemic control in these patients [41].

In contrast to the aforementioned benefits, the TZDs, in particular pioglitazone increased body weight (Table 3) [1, 18, 19, 25, 31], in part because of differentiation of adipocytes and expansion of adipocyte mass. Activation of PPAR-γ stimulates differentiation to insulin-sensitive smaller adipocytes and redistributes fat from visceral to subcutaneous depots, a pattern that has been associated with lower cardiovascular disease (CVD) risk. On the other hand, DPP-4 inhibitors have generally neutral effect on weight [1, 23, 31]. It was found out that there was no weight gain with any DPP-4 inhibitors [SAXA: 0.06 (95 % CI−0.45, 0.57) SIT: 0.21 (95 % CI−0.1, 0.53) VIL: 0.04 (95 % CI−0.37, 0.44)], but TZDs were associated with significant increases in body weight [PIO: 2.06 (95 % CI 1.31, 2.81)] in a recent random-effects network meta-analysis, which included 62 randomized clinical trials (n = 32 185 participants) [42].

**Table 3:** Comparative table of clinical trials and studies, investigating the safety of treatment with gliptins and glitazones

Author	Treatment	Change in mean body weight
Bolli et al., 2009 [31]	VIL + MET	↑ 0.2 kg, non-significant
	PIO + MET	↑ 2.6 kg, *P* < 0.001
Jindal et al., 2015 [1]	VIL + MET	No change in body weight
	PIO + MET	↑1.2 ± 0.5 kg
Chawla et al., 2013 [25]	SIT + MET	↓ 0.58 kg, statistically significant
	PIO + MET	↑0.90 kg, statistically significant
Russell-Jones et al., 2012 [18]	EQW	↓ 2.0 kg
	MET	↓ 2.0 kg (*P* = 0.892 vs. EQW)
	PIO	↑ 1.5 kg (*P* < 0.001 vs. EQW)
	SIT	↓ 0.8 kg (*P* < 0.001 vs. EQW)
Rosenstock et al., 2007 [19]	VIL	↑ 0.2 ± 0.3 kg
	PIO	↑ 1.5 ± 0.3 kg
	VIL + PIO (50/15 mg)	↑ 1.4 ± 0.3 kg
		↑ 2.1 ± 0.3 kg
	VIL + PIO (100/30 mg)	
		Risk of fractures
Bone et al., 2013 [50]	PIO	BMD of total proximal femur (primary and point):
	PLB	Least squares mean from baseline: −0.69 % PIO, −0.14 % PLB (*P* = 0.170) statistically non-significant
		Bone remodeling markers:
		statistically non-significant between-group differences
Bazelier et al., 2012 [46]	Biguanide or Sulfonyluerum	↓ risk HR = 1.15, 95 % CI 1.13–1.18
	Biguanide and Sulfonyluerum	↓ risk HR = 1.00, 95 % CI 0.96–1.04
	TZDs	↑ risk HR = 1.27, 95 % CI 1.06–1.52
	Insulin	↑ risk HR = 1.25, 95 % CI 1.20–1.31
Glintborg et al., 2008 [47]	PIO	↓BMD [geometric means (−2 to +2_SD_): lumbar spine 1.140 (0.964–1.348) vs. 1.127 (0.948–1.341)g/cm^2^ (average decline 1.1 %) and femoral neck 0.966 (0.767–1.217) vs. 0.952 (0.760–1.192)g/cm^2^ (average decline 1.4 %), both *p* < 0.05]
	PLB	
Meier et al., 2008 [48]	PIO	↑ hip and nonvertebral osteoporotic fractures OR = 2.59, 95 % CI 0.96–7.01
	ROSI	↑ hip and nonvertebral osteoporotic fractures OR = 2.38, 95 % CI 1.39–4.09
Colhoun et al., 2012 [49]	PIO	↑hip fractures risk OR = 1.18, 95 % CI 1.00–1.40
	ROSI	↑hip fractures risk OR = 1.16, 95 % CI 1.06–1.26
Bunck et al., 2012 [53]	VIL	Bone resorption marker: S-CTx (cross-linked C-terminal telopeptide): between-group ratio 1.15 ± 0.17, *P* = 0.320 serum alkaline phosphatase, calcium and phosphate - unaffected
	PLB	
Monami et al., 2011 [52]	DPP-4	↑ risk of bone fractures Mantel Haenszel odds ratio [MH-OR] 0.60, 95 % CI 0.37–0.99, *P* = 0.045
	PLB, other treatments	
		Risk of cardiovascular complications
Dormandy et al., 2005 [55]	PIO	↓ all-cause mortality, non-fatal myocardial infarction, and stroke (main secondary endpoint)
		↑ HF (11 % vs. 8 %, *p* < 0.0001)
	PLB	
Wilcox et al., 2007 [56]	PIO	↓ fatal or nonfatal stroke (HR = 0.53, 95 % CIs = 0.34–0.85; *P* = 0.0085)
		↓ cardiovascular death, nonfatal myocardial infarction, or nonfatal stroke (HR = 0.72, 95 % CIs = 0.53–1.00; *P* = 0.0467)
	PLB	
Nissen et al., 2007 [57]	ROSI	↑ myocardial infarction (OR = 1.43, 95 % CI, 1.03–1.98; *P* = 0.03)
		↑ death from cardiovascular causes (OR = 1.64, 95 % CI, 0.98–2.74; *P* = 0.06)
	Control group	
Lincoff et al., 2007 [59]	PIO	↓ death, myocardial infarction, or stroke (OR = 1.43, 95 % CI, 1.03–1.98; *P* = 0.03)
		↑ HF (НR, 1.41; 95 % CI, 1.14–1.76; *P* = 0.002)
	Control group	
Gallagher et al., 2011 [63]	PIO	
	ROSI	↑ death (RR 1.20; 95 % CI 1.08–1.34)
		↑ HF (RR 1.73; 95 % CI 1.19–2.51)
Breunig et al., 2014 [62]	PIO	
	ROSI	↑ HF (HR = 1,79, 95 % CI = 1.16–2.76, *P* = 0.009)
	MET	
Seong et al., 2015 [61]	PIO + MET	↓risk of CVD 0.89 (95 % CI, 0.81–0.99)
		↓risk of IS 0.81 (95 % CI, 0.67–0.99)
		↑risk of HF 4.81 (95 % CI, 3.53–6.56)
	DPP-4i + MET	
Scirica et al., 2013 [65]	SAXA	↑ HF (HR 1.27; 95 % CI, 1.07–1.51; *P* = 0.007)
Scirica et al., 2014 [66]	PLB	
Monami et al., 2014 [67]	DPP-4 inhibitors	↑ HF (MH-OR: 1.19[1.03; 1.37]; *p* = 0.015).
	Control group	
Clifton P, 2014 [68]	DPP-4 inhibitors	↑ HF
	Control group	
Wang et al., 2014 [69]	SIT	↑ HF (HR: 1.09, 95 % CI: 1.06–1.11, *P* < 0.001).
	Control group	
Chen et al., 2014 [70]	SIT	↑ recurrent myocardial infarction (HR, 1.73; 95 % CI, 1.15–2.58; *P* = 0.008)
		↑ percutaneous coronary revascularization (HR, 1.43; 95 % CI, 1.04–1.95; *P* = 0.026)
	Control group	
		Effects on liver
Belfort et al., 2006 [72]	PIO	↓alanine transaminase (by 58 % vs. 34 %, *P* < 0.001)
		↑hepatic insulin sensitivity (by 48 % vs. 14 %, *P* = 0.008)
		↓liver fat (by 54 % vs. 0 %, *P* < 0.001)
		↓liver inflammation (*P* = 0.008)
		↓ballooning necrosis (*P* = 0.02)
		Fibrosis not improved (*P* = 0.08)
	PLB	
Aithal et al., 2008 [73]	PIO	↓ hepatocellular injury (*P* = 0.005)
		↓Mallory-Denk bodies (*P* = 0.004)
		↓ alanine aminotransferase level (−10.9 vs −36.2 u/L; *P* = 0.009)
		↓ gamma-glutamyltransferase level (−9.4 vs −41.2 u/L; *P* = 0.002)
		↓ ferritin (−11.3 vs −90.5 μg/L; *P* = 0.01)
		Fibrosis improved (*P* = 0.05)
	PLB	
Sanyal et al., 2010 [74]	PIO	↓ serum alanine and aspartate aminotransferase levels (*P* < 0.001)
		↓ insulin resistance (*P* = 0.03)
		↓ liver inflammation (*P* = 0.004)
		↓ ballooning necrosis (*P* = 0.08)
		Fibrosis not improved (*P* = 0.12)
	PLB	
Ohki et al., 2012 [77]	SIT	↓ aspartate aminotransferase (*P* = 0.47)
		↓ alanine aminotransaminase (*P* = 0.03)
		↓ gamma-glutamyltransferase (*P* = 0.01)
	PIO	↓ aspartate aminotransferase (*P* < 0.01)
		↓ alanine aminotransaminase (*P* < 0.01)
		↓ gamma-glutamyltransferase (*P* < 0.01)
Iwasaki et al., 2011 [75]	SIT	↓ alanine transaminase, aspartate aminotransferase, gamma-glutamyltransferase
Itou et al., 2012 [76]	SIT – case report	↓ alanine transaminase, aspartate aminotransferase
		↓ insulin resistance
		↓ liver fat
		Risk of development of oncological disease
Azoulay et al., 2013 [2]	PIO	↑ bladder cancer (RR 1.83, 95 % CI 1.10–3.05)
Wei et al., 2013 [87]	PIO	↓ bladder cancer (HR, 1.16, 95 % CI 0.83, 1.62)
	Active control	
Govindarajan et al., 2007 [34]	PIO	↓lung cancer (RR, 0.67; 95 % CI, 0.51–0.87)
	Active control	
Mazzone et al., 2012 [56]	TZDs	↓ lung cancer (OR 0.86, 95 % CI 0.4–1.85, *p* = 0.14)
	MET	↓ lung cancer (OR 0.48, 95 % CI 0.28–0.81, *p* = 0.006)
Nelson et al., 2014 [31]	SIT - case report	↑ pancreatitis
Girgis and Champion, 2011 [81]	VIL - case report	↑ acute pancreatitis
Singh et al., 2013 [27]	EQW/SIT	↑acute pancreatitis (OR 2.01, 95 % CI [1.37–3.18], *P* = 0.01)
Engel et al., 2013 [45]	SIT Comparator agent	Rates of malignancy (−0.05 (95 % CI −0.41, 0.30)
		Rate of category of pancreatic cancer (adenocarcinoma of pancreas, pancreatic carcinoma, pancreatic carcinoma metastatic) (0.05 and 0.06 events per 100 patient-years in the sitagliptin and nonexposed groups, respectively)

*ROSI* rosiglitazone, *SAXA* saxagliptin, *АLO* alogliptin, *BMD* bone mineral density, *CVD* cardiovascular disease, *IS* ischemic stroke

New or worsening peripheral edema is common with TZD use, ranging from 2.5 % to 16.2 % incidence, with risk increasing with age, increasing drug dose, female sex, declining renal function, and concomitant insulin use [39, 40]. Typical, but manageable, increases in oedema (26.4 % vs 15.1 % for placebo) and weight gain (mean change of +3.8 kg vs−0.6 kg for placebo) associated with pioglitazone therapy in PROactive were reviewed by Dormandy et al. [43].

Current drug labels for TZDs warn of increased fractures, predominantly for distal fractures in women [44–46]. A higher rate of bone fractures was observed among pioglitazone-treated female patients (5.1 % vs 2.5 %) in PROactive study population [43]. Moreover, a randomized, placebo-controlled trial demonstrated that pioglitazone treatment was followed by decreased lumbar and hip bone mineral density (BMD) and decreased measures of bone turnover in premenopausal patients with polycystic ovary syndrome [47]. The findings of a large, population-based, nested, case–control analysis have provided further evidence that current use of rosiglitazone and pioglitazone in women and men with T2DM may be associated with an approximately 2- to 3-fold increased risk of hip and nonvertebral osteoporotic fractures [48]. Colhoun et al. observed hip fractures in both sexes and the risk was similar for pioglitazone users and rosiglitazone users [49]. Furthermore, not all studies have demonstrated such an increase in risk. According to Bazelier et al., risk of osteoporotic fracture was similar for TZD users and insulin users, but versus nondiabetic patients TZD users showed a 1.3-fold increased risk of fracture. In their opinion the underlying T2DM should be taken into account, when studying fracture risk with TZDs [46]. The results of a prospective, double-blind study did not demonstrate a causal relationship between pioglitazone treatment for 12 months and loss of bone mass or alteration of bone remodeling that would be expected to result in excessive bone fragility [50]. The effect of TZDs on bone is a drug class effect and duration of treatment is proportional with increased fracture risk [51]. Preclinical studies have suggested that incretin-based therapies may be beneficial for the bone; however, clinical data are largely lacking. A meta analysis performed by Monami et al. on all trials that enrolled T2DM patients that received DPP-4 inhibitors for at least 24 weeks suggested that DPP-4 inhibitors could be associated with reduced risk of bone fracture [52], but the duration of included trials was short and bone fracture were not the principal end points in any of the studies and were reported only as adverse events, and were probably not described carefully. A neutral role of DPP-4 inhibitors on bone metabolism was demonstrated by treatment with vildagliptin (100 mg once daily) in drug naïve type 2 diabetic patients for 1 year. Circulating levels of markers of bone resorption (cross-linked C-terminal telopeptide) and calcium homeostasis (serum alkaline phosphatase, calcium, and phosphate) were unaffected compared to baseline and to placebo [53]. However, clinical evidence for DPP-4 inhibitors in humans is still not enough to allow definitive conclusions and further studies are required for their long-term efficacy and safety on bone metabolism.

Pioglitazone carries black box label warnings about its potential to cause or exacerbate congestive heart failure and is contraindicated in patients with New York Heart Association class III or IV heart failure (HF) [54]. Pioglitazone is the only hypoglycemic drug, other than metformin, with conducted randomized trial which has clearly demonstrated that it reduces mortality, myocardial infarction and stroke. PROactive is a large, prospective, randomized, double-blind study that examined the effect of pioglitazone (45 mg /day) on macrovascular complications in 5238 patients with T2DM and concomitant cardiovascular diseases. The study showed that pioglitazone non-significantly reduces (10 %) the risk of the composite primary endpoint— death from any cause, non-fatal myocardial infarction (including silent myocardial infarction), stroke, acute coronary syndrome, leg amputation, coronary revascularisation, or revascularisation of the leg. The pre-defined main secondary endpoint—all-cause mortality, myocardial infarction, or stroke—was also significantly reduced, in the pioglitazone group. Despite the increase in reported heart failure in the pioglitazone group, the number of deaths from heart failure was similar in each group [55].

Pioglitazone is beneficial in reducing the chances of a patient who has had a stroke from having further stroke. A subgroup analysis from PROactive study found that pioglitazone reduced the occurrence of fatal or non-fatal stroke (5.6 % in the pioglitazone group against 10.2 % in the placebo group) and mortality due to cardiovascular complications, non-fatal myocardial infarction or non-fatal stroke (13.0 % in the pioglitazone group against 17.7 % in the placebo group) compared with placebo [56].

Whilst a meta-analysis has raised concerns regarding the possibility of cardiovascular complications associated with rosiglitazone [57, 58], another meta-analysis of pioglitazone trials showed that among the diverse population of patients with T2DM, treatment with pioglitazone was associated with a significant decrease in risk of myocardial infarction, stroke or death [59, 60]. The results of a population-based cohort study showed that pioglitazone + metformin was associated with decreased risks of total cardiovascular disease and ischemic stroke compared with DPP-4 inhibitor + metformin. However, the risk of HF was higher in patients receiving pioglitazone + metformin [61]. Compared with metformin the risk of developing HF in Medicaid (US government insurance program) patients was higher in patients started on rosiglitazone, but not pioglitazone [62]. The study, which supported the suspension of rosiglitazone by European regulatory authorities in September 2010, determined that current rosiglitazone users had an increased risk of death (adjusted RR 1.20; 95 % CI 1.08–1.34) and hospitalization for HF (adjusted RR 1.73; 95 % CI 1.19–2.51) compared to current pioglitazone users [63]. The difference between rosiglitazone and pioglitazone in regard to cardiovascular complications suggests that these effects are rather associated with the TZD type, but are not a drug class effect. It was found that pioglitazone is associated with greater PPAR-γ activation than rosiglitazone [64]. A retrospective review showed that treatment with pioglitazone was associated with greater beneficial effects on blood lipid levels (triglycerides, total cholesterol, and LDL-C) than rosiglitazone [34]. Therefore, differential therapeutic modulation of lipid levels might confer a different level of protection from cardiovascular disease in patients with T2DM.

T2DM and HF often coexist, and together, these conditions are associated with increased morbidity and mortality compared with each of them individually. The cardiovascular safety and efficacy of pioglitazone is proven, while this was not done with inhibitors of DPP-4. Recently (2013), cardiovascular safety and efficacy of saxagliptin was estimated in 16 492 patients with T2DM and concomitant cardiovascular risk factors in the study - Saxagliptin Assessment of Vascular Outcomes Recorded in Patients With Diabetes Mellitus (SAVOR) - Thrombolysis in Myocardial infarction (TIMI) 53. Scirica et al. analyzed in detail the risk of occurrence of HF in this study. It was found that more patients in the saxagliptin group are hospitalized due to HF (3.5 % vs. 2.8 %; HR, 1.27; 95 % CI, 1.07 to 1.51; *P* = 0.007) and this DPP-4 inhibitor did not provide any cardioprotective benefit [65, 66]. In a meta-analysis that included 84 studies it was established that the overall risk of HF was higher in patients treated with DPP-4 inhibitors compared with placebo/active control without any clear evidence of differences among drugs of the class [67]. Data from another meta-analysis revealed that in the randomized, controlled trials (including sitagliptin, saxagliptin and alogliptin) the risk of HF in patients using DPP-4 inhibitors was 24 % [68].

The available evidence suggests that the DPP-4 inhibitors are associated with increased risk of HF [67–69] as well as they have unproven safety with respect to the cardiovascular system. Patients with T2DM, chronic kidney disease and acute myocardial infarction were included in a cohort study which demonstrated that using sitagliptin increased the risk of recurrent myocardial infarction and percutaneous coronary revascularization [70].

In 2008 the Food and Drug Administration (FDA) in the United States required that the cardiovascular safety for all glucose lowering drugs was proven, and this began to be applied to new antidiabetic drugs: DPP-4 inhibitors. In 2013–2014 many studies with DPP-4 inhibitors were conducted, which have definitely shown a higher risk of developing HF and the cardiovascular safety of these inhibitors has to be demonstrated. On the other hand, the benefits and safety of pioglitazone on the cardiovascular system have been well established. Recently, post-marketing trials of DPP-4 inhibitors have shown that these drugs neither reduce nor increase the risk of major cardiovascular events compared with placebo [65, 71]. The safety of gliptins regarding the cardiovascular system has not been established in the long term.

Another advantage of pioglitazone, which characterizes its safe and efficient profile is its effect on liver histology, and hence its use for treatment of non-alcoholic fatty liver disease. Several studies have shown that the use of pioglitazone leads to reduced levels of liver enzymes and inflammatory markers of necrosis and improvements of fibrosis, steatosis and insulin sensitivity [72–74]. Several clinical studies with sitagliptin in subjects with T2DM and nonalcoholic steatohepatitis have shown decreases in alanine aminotransferase levels and improved liver histology [75–77]. Clinical data for the DPP-4 inhibitors is very scarce and the information is derived from non-randomized studies conducted in small groups of patients; this is why it is difficult to make final conclusions [78, 79].

Definitive data in humans, associating the TZDs with cancer development is not available. Azoulay et al., reported an increased risk of bladder cancer in the group of patients using pioglitazone (HR 1.83; 95 % CI 1.10–3.05) [80], while Wei et al., analyzed the risk in 23,548 patients exposed to pioglitazone and 184,166 exposed to other antidiabetic treatments and reported that pioglitazone may not be significantly associated with an increased risk of bladder cancer (1.16; 95 % CI 0.83–1.62) [81, 82]. The largest study examining the side effects of pioglitazone - PROactive detected more cases of bladder cancer in the pioglitazone group, although the difference was not statistically significant. But in the same trial statistically significant reduction of cases with breast cancer in the pioglitazone group was observed. Other studies have demonstrated the protective effect of TZDs against the development of lung cancer in patients with diabetes [83, 84]. The Agency’s Committee for Medicinal Products for Human Use (CHMP) concluded that, although there is a small risk of bladder cancer with pioglitazone, its benefits continue to outweigh its risks in a limited population of type 2 diabetes patients (www.ema.europa.eu - Accessed on 21/7/201). On the other hand, data from studies, warnings of the European Medicines Agency (EMA) and the FDA have shown that there is increased risk of developing acute pancreatitis and pancreatic cancer in therapy with incretin-based agents: exenatide, liraglutide, sitagliptin, saxagliptin, vildagliptin, alogliptin and linagliptin. A lot of conflicting data [85–89] concerning the serious side effects of incretin-based therapies is currently available and published each month. Nevertheless, the widespread use of these drugs, the evaluation of short- and long-term risks should be considered as a priority [90].

Based on these facts, the balance between benefits and risks supports the use of pioglitazone to reduce the morbidity and mortality associated with T2DM.

### Costs comparison

In order to determine the benefits in terms of costs, the defined daily dose, the monthly cost of the treatment course and the level of reimbursement by the National Health Insurance Fund (NHIF) should be taken into account. The defined daily dose (DDD) for pioglitazone is 30 mg, and for sitagliptin and vildagliptin – 100 mg. According to the updated PDL the reimbursement rate for pioglitazone is 25 %. In comparison, sitagliptin and vildagliptin are reimbursed at 100 % by NHIF, despite their much higher wholesale price. Table 4 provides the reference prices of mono and combination therapies with the three antihyperglycemic drugs. All prices are given in BGN and EUR with current exchange rates of 1.95583 BGN for 1 EUR according to Bulgarian National Bank.

**Table 4:** Cost of monthly therapy

Product	DDD, mg	Cost of DDD,	Cost of monthly therapy,	Relative difference in the
		BGN (EUR)	BGN (EUR)	cost versus PIO or PIO + MET, %
PIO	30	1.56156 (0.79841)	46.84 (23.95)	-
SIT	100	2.64929 (1.35456)	79.48 (40.64)	+70
VIL	100	2.73429 (1.39802)	82.03 (41.94)	+75
PIO + МET 15/850 mg	30/2000	1.68522 (0.86164)	50.56 (25.85)	-
PIO + МET 15/1000 mg	30/2000	1.68522 (0.86164)	50.56 (25.85)	-
SIT + MET 50/850 mg	100/2000	3.18204 (1.62695)	95.46 (48.81)	+89
SIT + MET 50/1000 mg	100/2000	3.20408 (1.63822)	96.12 (49.15)	+90
VIL + МET 50/850 mg	100/2000	3.24533 (1.65931)	97.36 (49.78)	+93
VIL + MET 50/1000 mg	100/2000	3.08333 (1.57407)	92.50 (47.29)	+83

Given that the effectiveness of pioglitazone is comparable or even better than that of gliptins and it has shown better safety than them, then the exchange of mono- and combination therapy with gliptins with monotherapy and combination therapy with pioglitazone will allow savings of public funds to be realized by NHIF.

Data on the amount of sold packs of sitagliptin, vildagliptin and their combination with metformin has been taken from IMS Health database. It was estimated that if sitagliptin or vildagliptin monotherapy was exchanged with pioglitazone monotherapy and it was reimbursed by 100 % 682,217.76 BGN (348,812.40 EUR) and 90,437.24 BGN (46,239.83 EUR) will be saved, respectively (Table 5).

**Table 5:** Effectiveness of annual costs during treatment with pioglitazone (monotherapy and combination therapy) in Bulgaria

Treatment	Number of packs sold in 2014	Expenses, BGN (EUR)	Number of patients taking DDD	Cost of therapy with PIO or PIO + MET, BGN (EUR)	Difference, BGN (EUR)	Amount reimbursed by the NHIF, BGN (EUR)
SIT	25,072	1,859,840.96 (950,921.58)	25,072	1,177,623.20 (602,109.18)	682,217.76 (348,812.40)	682,217.76 (348,812.40)
VIL	6,088	233,048.64 (119,155.88)	3,044	142,611.40 (72,916.05)	90,437.24 (46,239.83)	90,437.24 (46,239.83)
SIT + MET 50/850 mg	141,986	6,325,476.30 (3,234,164.68)	70,993	3,588,696.15 (1,834,871.21)	2,736,780.15 (1,399,293.47)	1,368,390.07 (699,646.73)
SIT + MET 50/1000 mg	257,550	11,553,693.00 (5,907,309.43)	128,775	6,508,288.50 (3,327,634.81)	5,045,404.50 (2,579,674.36)	2,522,702.25 (1,289,837.18)
VIL + MET 50/850 mg	53,980	5,255,492.80 (2,687,090.80)	26,990	1,364,344.50 (697,578.27)	3,891,148.30 (1,989,512.53)	1,945,574.15 (994,756.27)
VIL + MET 50/1000 mg	128,665	11,901,512.50 (6,085,146.72)	64,332.5	3,251,364.55 (1,662,396.30)	8,650,147.95 (4,422,750.42)	4,325,074.98 (2,211,375.72)
Total:	613,341	37,129,064.20 (18,983,789.08)	319,206.5	16,032,928.30 (8,197,183.96)	21,096,135.90 (10,786,283.01)	10,934,395.45 (5,590,649.35)

Therefore, if therapies with sitagliptin + metformin and vildagliptin + metformin are replaced with pioglitazone + metformin and reimbursed at 50 %, the NHIF would realize savings for BGN 10,161,740.45 (5,195,615.39 EUR).

Our results are confirmed by Klarenbach et al., who used the United Kingdom Prospective Diabetes Study Outcomes Model to predict diabetes-related complications, quality-adjusted life-years (QALY) and costs of alternative second-line therapies available in Canada for adults with T2DM inadequately controlled by metformin. DPP-4 inhibitors, basal insulin and biphasic insulin are more costly and lead to fewer QALYs than TZDs, they are dominated by the TZDs (which are less expensive and more effective) [91].

An economic modeling analysis carried out in the USA suggested that pioglitazone may deliver superior economic value when compared to sitagliptin due to improved HbA_1c_ and cardiovascular outcomes at reasonable incremental cost [92].

## Conclusion

Pioglitazone has a glycemic profile which is superior or similar to that of newer antidiabetic drugs (DPP-4 inhibitors). It does not increase the risk of cardiovascular complications and has a manageable safety profile. At present the commonly used therapies with newer drugs (sitagliptin and vildagliptin) seem to be both less effective and less safe. Furthermore, none affect insulin sensitivity as beneficially as pioglitazone. In addition to its favorable effect on glycemic parameters pioglitazone proves to be cost-effective when compared to sitagliptin and vildagliptin. Nevertheless, gliptins have a growing market share, despite the lack of data on long-term safety and their higher price. If the drug and reimbursement policy in Bulgaria follows the principles for reimbursement of cost-effective treatments and gives preference to pioglitazone, it could save up to BGN 10,934,395.45 (5,590,649.35 EUR).

## Abbreviations

T2DM: Type 2 diabetes mellitus
TZD: Thiazolidinediones
PPARs: Peroxisome proliferator-activated receptors
DPP-4: Dipeptidyl peptidase-4
NICE: National Institute for Health and Care Excellence
HbA_1c_: Glycated hemoglobin
PDL: Positive drug list
FPG: Fasting plasma glucose
HDL: High-density lipoprotein
LDL: Low-density lipoprotein
HF: Heart failure
FDA: Food and drug administration
EMA: European medicines agency
NHIF: National health insurance fund
DDD: Defined daily dose
QALY: Quality-adjusted life-years
